# ACTN3 Polymorphism: Comparison Between Elite Swimmers and Runners

**DOI:** 10.1186/s40798-015-0023-y

**Published:** 2015-06-14

**Authors:** Sigal Ben-Zaken, Alon Eliakim, Dan Nemet, Moran Rabinovich, Eias Kassem, Yoav Meckel

**Affiliations:** 1 Genetics and Molecular Biology Laboratory, The Zinman College of Physical Education and Sports Sciences at the Wingate Institute, Netanya, 42902 Israel; 2 Child Health and Sports Center, Pediatric Department, Meir Medical Center, Sackler School of Medicine, Tel-Aviv University, Tel-Aviv, Israel; 3 Pediatric Department, Hillel-Yafe Medical Center, Hadera, Israel

**Keywords:** ACTN3, Genetic polymorphism, Runners, Swimmers

## Abstract

**Background:**

The human *ACTN3* gene encodes α-actinin-3, an actin-binding protein with a pivotal role in muscle structure and metabolism. A common genetic single nucleotide polymorphism (SNP) at codon 577 of the *ACTN3* results in the replacement of an arginine (R) with a stop codon (X). The R allele is a normal functional version of the gene, whereas the X allele contains a sequence change that completely stops production of functional α-actinin-3 protein. The *ACTN3* R577X polymorphism was found to be associated with power athletic performance especially among track and field athletes. The aim of the current study was to compare allelic and genotype frequencies of the ACTN3 R577X polymorphism among runners and swimmers specializing in different distances, and >non-athletic controls.

**Methods:**

One hundred and thirty-seven runners, 91 swimmers and 217 controls, participated in the study. Runners were assigned to two subgroups according to their event specialty—long-distance runners (LDR) and short-distance runners (SDR). Swimmers were also assigned to two subgroups according to their main swimming event—long-distance swimmers (LDS) and short-distance swimmers (SDS). Genomic DNA was extracted from peripheral EDTA-treated anti-coagulated blood using a standard protocol. Genotypes were determined using the Taqman allelic discrimination assay.

**Results:**

Runners’ genotype and allele differed significantly between LDR, SDR, and controls, with the lowest prevalence of RR genotype and R allele among LDR. XX genotype and X allele prevalence was significantly higher among LDR compared to the other groups (*p* < 0.01 for all). On the other hand, swimmers’ genotype and allele frequencies did not differ significantly between subgroups (LDS and SDS). Yet, LDS had significantly higher RR genotype and R allele frequencies compared to LDR.

**Conclusions:**

The findings suggest that while ACTN3 R577X polymorphism is a genetic polymorphism that may distinguish between SDR and LDR, it cannot differentiate significantly between SDS and LDS.

**Trial Registration:**

ClinicalTrials.gov: NCT01319032

**Key Points:**

- *ACTN3* R577X polymorphism is largely associated with running events specialization, with high prevalence of RR genotype and R allele frequency among short-distance runners compare to long-distance runners.
- Unlike in running, *ACTN3* R577X polymorphism is not associated with swimming specialization.
- The inability of the *ACTN3* R577X polymorphism to distinguish between swimmers specializing in different events, presumably since other factors such as body physique, technique, tactics, etc., are more likely to determine such a distinction.

## Background

Athletic performance is a multifactorial trait, determined by a range of genetic and environmental factors. Most of the genetic polymorphisms considered to be related to athletic performance were investigated in case-control retrospective studies [1], which did not reveal the biological mechanism responsible for the influence of this polymorphism on athletic performance. Moreover, most of these polymorphisms are intronic and non-functional.

The *ACTN3* R577X (rs1815739) polymorphism is an exception in this sense, since it is a well-studied functional polymorphism. The human *ACTN3* gene encodes α-actinin-3, an actin-binding protein with a structural role at the sarcomeric Z-line in glycolytic (type II, fast-twitch) muscle fibers, and plays an increasingly evident role in the regulation of muscle metabolism [2]. A common genetic single nucleotide polymorphism (SNP) at codon 577 of the *ACTN3* results in the replacement of an arginine (R) with a stop codon (X) [3]. The R allele is a normal functional version of the gene, whereas the X allele contains a sequence change that completely stops production of functional α-actinin-3 protein [3]. Therefore, XX homozygotes do not express ACTN3 at all in their muscles [2]. In the knockout mouse, it is clear that ACTN3 deficiency alters skeletal muscle function [2].

Yang et al. [4] demonstrated, for the first time, a significant association between the ACTN3 genotype and athletic performance. They found that both male and female elite sprint athletes have significantly higher frequencies of the 577R allele compared to controls. Since then ACTN3 R577X has been studied in various cohorts. Some articles have reported a strong association between the RR genotype and elite power performance [5–8]. While ACTN3 R carriage may enhance power performance, ACTN3 XX might contribute to endurance performance [9, 10]. However, reports on Asians and Africans suggested that ACTN3 deficiency might not be associated with endurance performance [11, 12]. A recent meta-analysis of 88 articles did not find a significant association for ACTN3 RR genotype with physical performance (odds ratio (OR), 1.03; 95 % confidence interval (CI), 0.92–1.15). However, when the analysis was restricted to power events, a significant association was observed (OR, 1.21; 95 % CI, 1.03–1.42) [13]. Overall, association studies on ACTN3 R577X polymorphism and power performance show consistent results across multiple athlete cohorts [14]. Moreover, unlike many polymorphisms, the ACTN3 R577X polymorphism is a functional polymorphism, with the XX genotype resulting in complete loss of function. A mechanistic explanation for the role of ACTN3 in power performance can be found in the α-actinin-3 knockout (KO) mouse. Compared with wild-type mice, the muscles of the KO mouse exhibit reduced muscle mass, due to decreased diameter of fast muscle fibers, significant decrease in grip strength, higher endurance, and a shift towards increased activity of mitochondrial oxidative metabolism [9, 10]. All of these placed the ACTN3 R577X polymorphism as a pivotal polymorphism explaining power athletic performance [14].

Interestingly, while the ACTN3 R577X polymorphism has been studied extensively among track and field athletes (especially runners), its contributions to performance in swimming, a sport that muscle strength plays also a key role [15], had received little attention. In swimming, muscular strength dictates how much force muscles are able to apply to the water, which in turn propels the body forward. Therefore, muscular strength is of great importance in order to produce speed in sprint swimming, while in endurance swimming muscular strength is required to perform repeated submaximal contractions over time. The few studies that investigate ACTN3 R577X prevalence among swimmers did not find significant association between the ACTN3 R577X polymorphism and swimming performance among Caucasian, East Asians [16], or Spanish swimmers [17]. Among Taiwanese, the R allele was significantly higher in female international sprint swimmers than in national sprint swimmers or the general population [5].

Therefore, the aim of the current study was to compare allelic and genotype frequencies of the ACTN3 R577X polymorphism among runners and swimmers specializing in different distances, and non-athletic controls. We hypothesized that similarly to runners, the R allele will be more frequent among sprint swimmers and the X allele more frequent among endurance swimmers.

## Methods

### Participants

One hundred and thirty-seven track and field athletes (98 males and 39 females, age 17–50) and 91 swimmers (60 males and 31 females, age 16–49) participated in the study. The track and field athletes were assigned to two subgroups according to their event specialty, as follows: 1) long-distance runners (5000 m marathon runners—LDR) (*n* = 65) and 2) power event athletes (100–200 m sprinters and long jumpers—SDR) (*n* = 72). The swimmers were also assigned to two groups according to their main swimming event, as follows: 1) long-distance swimmers (800–1500 m swimmers—LDS) (*n* = 43) and 2) short-distance swimmers (50–100 m swimmers—SDS) (*n* = 48). All athletes were ranked among the top Israeli results in their event and had competed in national and/or international level meets on a regular basis. Sixty-seven track and field athletes were classified as international athletes (participants in European and World Championships, and Olympic Games). The Israeli runners’ criteria for participation in the Olympic Games and World championships are ranking in places 1–12 in the former European championship or 1–16 in the former world championship (both should be no more than 2 years prior to the upcoming event), or setting the international result criteria A at least once. For participation in a European championship, the runners should fulfill the above criteria or set the international result criteria B at least twice. The international results criteria A and B are standard results published yearly by the IAAF (The International Association of Athletic Federation, http://www.iaaf.org/). These standard results are intended to qualify athletes to be eligible to compete in Olympics and World Championships. The A standard is the most difficult to achieve. The B standard is easier to achieve, and usually, national athletic association uses it also as a criterion to international competition participation.

Thirty-one swimmers were classified as international athletes (participants in European and World Championships, and Olympic Games). The Israeli swimmers result criteria for participation in the European and World championships and in the Olympic Games are the average result of the 16th place in the former three respective championships. These criteria indicate a remarkable difference between the international and national level athletes in the present study. The control group consisted of 217 (137 males and 80 females, age 19–29) non-athletic healthy individuals who were not engaged in competitive sport. Characteristics of the athletes and controls are presented in Table 1.

**Table 1:** Athletes and controls’ data

Group	Number	Main event	M/F	Top/national level	Age (mean + SD, range)
Runners					
Long distance	65	5000 m marathon	50/15	21/44	31.4 + 9.2 (17–50)
Power event (sprinters and jumpers)	72	100–200 m, jumps	48/24	23/49	30.1 + 12.7 (17–50)
Swimmers					
Long distance	43	800–1500 m	27/16	15/28	23.6 + 7.9 (16–49)
Short distance	48	50–100 m	33/15	16/32	23.3 + 8.3 (16–48)
Controls	217	nr	137/80	nr	26.4 + 5.8 (19–29)

*nr* not relevant

The study was approved by the Institutional Review Board of the Hillel Yaffe Medical Center, Hadera, Israel, according to the Declaration of Helsinki. A written informed consent was obtained from all participants.

### Genotyping

Genomic DNA was extracted from peripheral EDTA-treated anti-coagulated blood using a standard protocol. Genotypes were determined using the Taqman allelic discrimination assay. The Assay-by-Design service (www.appliedbiosystem.com) was used to set up a Taqman allelic discrimination assay for the ACTN3 (rs1815739 C1747T). Primer sequences were as follows: forward, GCACGATCAGTTCAAGGCAAC; reverse, GCTGAGGGTGATGTAGGGATTG. Probe sequences were for C1747T: forward, VIC-CGAGGCTGACCGAGAG; reverse, FAM-CCGAGGCTGACTGAGAG. The PCR reaction mixture included 5 ng genomic DNA, 0.125 μl TaqMan assay (40*, ABI), 2.5 μl Master mix (ABI), and 2.375 μl water. PCR was performed in 96 well PCR plates in an ABI 7300 PCR system (Applied Biosystems Inc., Foster City, CA, USA) and consisted of initial denaturation for 5 min at 95  °C, and 40 cycles with denaturation of 15 s at 95  °C and annealing and extension for 60  s at 63 °C. The results were analyzed by the ABI Taqman 7900HT using the sequence detection system 2.22 software (Applied Biosystems Inc).

### Statistical Analysis

The SPSS statistical package, version 20.0, was used to perform all statistical evaluations (SPSS, Chicago, IL, USA). A chi-squared test was used to confirm that the observed genotype frequencies were in Hardy-Weinberg equilibrium and to compare alleles and genotype frequencies between athletes and controls, as well as between athletes from different sports (e.g., runners vs. swimmers) and different competitive groups (e.g., sprinters vs. endurance). If observed or expected values included a cell value of 5, we used Fisher’s exact test to compare alleles and genotype frequencies.

## Results

The complete data on allele and genotype frequencies are presented in Table 2. The genotype subtype did not differ by age or sex. The ACTN3 genotype distribution was in agreement with the Hardy-Weinberg equilibrium in all groups (*p* = 0.87 for LDR, *p* = 0.84 for SDR, *p* = 0.99 for LDS, *p* = 0.78 for SDS, and *p* = 0.13 for controls).

**Table 2:** The ACTN3 R577X genotype and allele frequencies in all groups

Group	Number	Genotype			Allele frequency	
		R/R	R/X	X/X	R allele	X allele
Runners						
Long distance	65	13(20.0)*,**	29(44.6)*,**	23(35.4)*,**	55(42.3)^¥^, ^¥¥^	75(57.7)^¥^, ^¥¥^
Short distance	72	29(40.3)***	31(43.1)***	12(16.7)***	89(61.8)^¥¥¥^	55(38.2)^¥¥¥^
Total	187	57(30.5)	86(46.0)	44(23.5)	200(53.5)	174(46.5)
Swimmers						
Long distance	48	18(37.5)^#, ^^	18(37.5)^#^	12(25.0)^#^	57(57.0)&	43(43.0)&
Short distance	43	16(37.2)^^	22(51.2)	5(11.6)	55(62.5)	33(37.5)
Total	91	34(37.4)	40(43.9)	17(18.7)	108(59.3)	74(40.7)
Controls	217	48(22.1)	129(59.4)	40(18.4)	225(51.8)	209(48.2)

Values are absolute (relative frequencies in parentheses)

*χ ^2^(2) = 8.50, *p* < 0.01, genotype frequency, LDR vs. controls

**χ ^2^(2) = 9.29, *p* < 0.01, genotype frequency, LDR vs. SDR

***χ ^2^(2) = 9.41, *p* < 0.01, genotype frequency, SDR vs. controls

^#^ *χ* ^2^(2) = 8.01, *p* < 0.02, genotype frequency, LDS vs. controls (*χ* ^2^(1) = 6.14, *p* < 0.01, RR genotype, SDS vs. controls

^*χ* ^2^(1) = 4.25, *p* < 0.04, RR genotype, LDR vs. LDS

^^*χ* ^2^(1) = 4.97, *p* < 0.03, RR genotype, SDS vs. controls

^¥¥^ *χ* ^2^(1) = 10.42, *p* < 0.001, allele frequency, LDR vs. SDR

^¥¥¥^ *χ* ^2^(1) = 4.32, *p* < 0.05, allele frequency, SDR vs. controls

&*χ* ^2^(1) = 4.30, *p* < 0.04, allele frequency, LDR vs. LDS

**Fig. 1:**
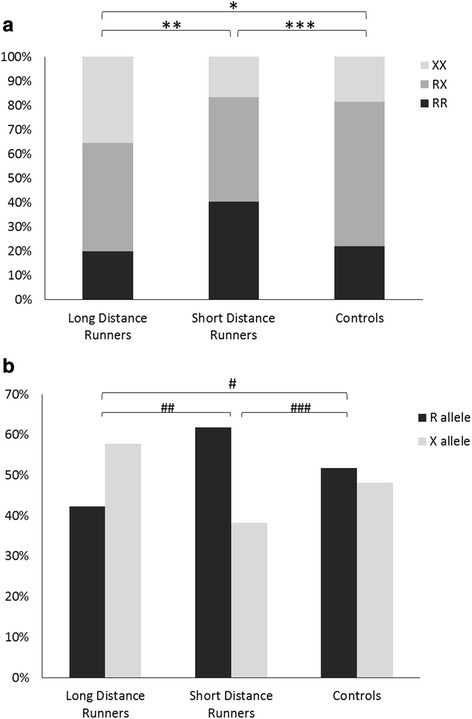
The ACTN3 R577X polymorphism **a** genotype frequencies, **b** allele frequencies in track and field athletes and controls. *χ ^2^(2) = 8.50, *p* < 0.01, genotype frequency, LDR vs. controls. **χ ^2^(2) = 9.29, *p* < 0.01, genotype frequency, LDR vs. SDR. ***χ ^2^(2) = 9.41, *p* < 0.01, genotype frequency, SDR vs. controls. #*χ* ^2^(1) = 3.64, *p* = 0.06, allele frequency, LDR vs. controls. ##*χ* ^2^(1) = 10.42, *p* < 0.001, allele frequency, LDR vs. SDR. ###*χ* ^2^(1) = 4.32, *p* < 0.05, allele frequency, SDR vs. controls

Runners’ genotype and allele frequencies are presented in Fig. 1. The runners’ genotype differed significantly between long-distance runners, short-distance runners, and controls (*p* < 0.01 for LDR vs. SDR, LDR vs. controls, and SDR vs. controls). The long-distance runners had the lowest frequency of RR genotype (20.0 % compared to 40 % among SDR). This frequency was similar to the RR genotype frequency among controls (22.1 %). On the other hand, the XX genotype frequency was significantly higher among LDR (35.4 %) compared to the other groups, SDR (16.7 %), and controls (18.4 %). LDR had a significantly higher frequency of X allele (57.7 %) compared to SDR (38.2 %, *p* < 0.01). SDR had significant higher R allele frequency (61.8 %) compared to LDR (42.3 %, *p* < 0.001) and controls (51.8 %, *p* < 0.05).

**Fig. 2:**
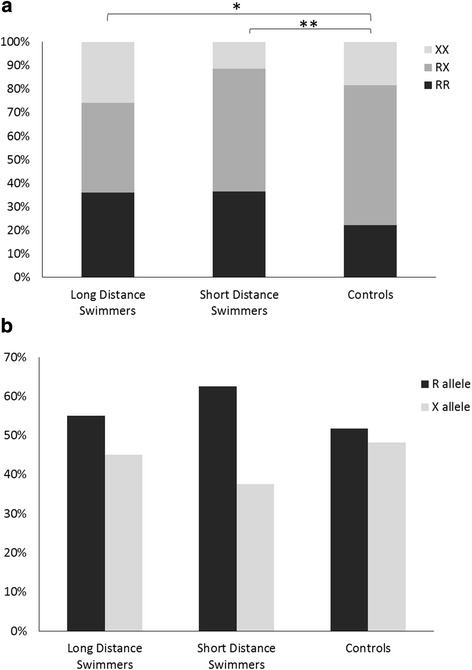
The ACTN3 R577X polymorphism (**a** genotype frequencies, **b** allele frequencies) in swimmers and controls. *χ ^2^(2) = 8.01, *p* < 0.02, genotype frequency, LDS vs. controls. **χ ^2^(1) = 4.97, *p* < 0.03, RR genotype, SDS vs. controls

The swimmers’ genotype and allele frequencies are presented in Fig. 2. The swimmers’ genotype and allele frequencies did not differ significantly between the specialization subgroups (LDS and SDS). Yet, the swimmers’ genotype frequency differed significantly from the controls (*p* < 0.05). LDS’ and SDS’ RR genotype frequencies (37.5 and 37.2 %, respectively) were significantly higher compared to controls’ RR genotype frequency (22.1 %, *p* < 0.01 for LDS vs. controls and *p* < 0.03 for SDS vs. controls). LDS had higher XX genotype frequency (18.7 %) compared to SDS (11.6 %), but this difference did not reach statistical significance.

**Fig. 3:**
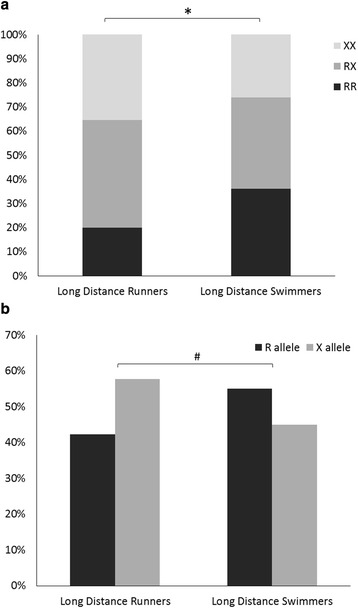
The ACTN3 R577X polymorphism (**a** genotype frequencies, **b** allele frequencies) in endurance athletes (LDR and LDS). *χ ^2^(1) = 4.25, *p* < 0.04, RR genotype, LDR vs. LDS. #*χ* ^2^(1) = 4.30, *p* < 0.04, allele frequency, LDR vs. LDS

We compared allele and genotype frequencies between athletes specializing in endurance-type events from different sports (e.g., LDS vs. LDR) and between athletes specializing in power-type events from different sports (e.g., SDS vs. SDR). LDS had significantly higher RR genotype and R allele frequency (37.4 and 59.3 %, respectively) compared to LDR (20.0 and 42.3 %, respectively) (*p* < 0.04 and *p* < 0.04 for RR genotype and R allele frequency, respectively) (Fig. 3). The power-type athletes’ genotype and allele frequencies did not differ significantly between SDR and SDS.

## Discussion

The main finding of the current study is that while the ACTN3 R577X polymorphism was largely associated with a distinction between runners specializing in sprint events compared to runners specializing in long-distance events, this difference was not found among swimmers specializing in sprint events compared to swimmers specializing in long-distance events.

Athletic events can be divided by the distance or time of the activity. Other parameters, like power, speed, and endurance, are also used to characterize specific sports. Track and field is a good example of the use of these descriptors and their activity time frames in differentiating between events based on energetic resources. Endurance-type athletic events are characterized by relatively low-intensity, long-lasting exercise that relies primarily on the aerobic energy-generating process [18]. The term aerobic refers to the use of oxidative phosphorylation to adequately meet energy demands during exercise [19]. In contrast, strength, power, and speed-type athletic events are characterized by intense efforts lasting a short time and by the use of anaerobic metabolism as the energy source.

The ACTN3 R577X polymorphism is a well-documented genetic marker that enables sport scientists to distinguish between a genetic predisposition towards excellence in power-type events or in endurance-type events [14]. Indeed, in the present study we found a significant difference in ACTN3 R577X polymorphism prevalence among LDR compared to SDR, with a high prevalence of 40.3 and 61.8 % for RR genotype and R allele frequency in SDR compared to 20.0 and 42.3 % for RR genotype and R allele frequency in LDR. This finding may suggest that the ACTN3 R577X polymorphism enables to distinguish between two types of track and field events—a “pure power event” and a “pure endurance event.” Yet, for a higher resolution distinction like the one needed to distinguish between middle-distance runners (MDR) and LDR performance, or between MDR and SDR performance, other genetic markers or profiles may be needed. This assumption should be examined in future studies. Since swimming events are also divided by the distance or time of the activity, one would assume that swimming events can also be classified into “endurance-type events” and “power-type events.” Subsequently, the genetic background that influences a track and field athlete’s capability to excel in one sport discipline (e.g., sprint running) rather than another (e.g., long-distance running) would, presumably, be similar to the genetic background that influences a swimmer’s capability to excel in a specific sport discipline. However, in contrast to our hypothesis, no significant association was found in the present study between the ACTN3 R577X polymorphism and swimming performance. This finding is consistent with previous reports that failed to find an association between the ACTN3 R577X polymorphism and swimming performance in Caucasians, East Asians [16], and Spanish swimmers [17]. In contrast, R allele was significantly higher in Taiwanese female international sprint swimmers compared to national-level sprint swimmers and the general population [5].

ACTN3 plays a pivotal role in muscle metabolism, structure, and fiber-type distribution [2, 20], and therefore it has a direct effect on the ability to perform in elite power events. However, based on the present findings, it may be that none of these are detrimental to swimming. It may be that the aspect of power performance affected by the polymorphism is less important in swimming relative to other sports, possibly because of the relatively lower stress put on muscles supported in water and the lack of eccentric contractions [21]. Moreover, in swimming the produced power is lower compared to land activities of similar duration [22]. Efficiency is a critical factor in this sport, which includes several aspects of technique. Swimming is a highly skilled sport, where the neural and biomechanical skills are the greatest contributor for force production [22–24]. Overall, in swimming, the athlete’s technique and body physique have a greater impact on performance than in other sports such as running.

In line with this, we have previously found a strong significant correlation (*r* = 0.74) between 100 m and the 2000 m swim times suggesting that swimming times are largely affected by swimming technique and by the swimmers’ size (particularly limb length) [25]. These may, at least partially, mask metabolic differences between swimmers, enabling technically skilled and/or tall swimmers to excel at all swimming distances. These relationships are unique for swimming, and this assumption can be supported by the past records of top world-class swimmers, such as Ian Thorpe (world record holder in the 100-m relay and individual 200, 400, and 800 m) and Grant Hackett (world record holder in 200-m relays and individual 400, 800, and 1500 m), as well as others who excel in both short and long swimming distances. A similar phenomenon is very uncommon among runners. To the best of our knowledge, this relationship between short- and long-distance performances in swimming has not been reported previously in other sport types.

Lastly, there is the possibility that a type II error accounts for the fact that we do not see an association between the ACTN3 R577X polymorphism and swimming performance. Large studies with the power to detect significant associations at genome-wide level have not yet been conducted. Although a meta-analysis of the association between ACTN3 and sprint/power athlete status demonstrated evidence for a real association [13, 26], many studies—mainly with small sample sizes—have failed to observe any association between ACTN3 variants and sporting performance.

It should be noted that both LDS and SDS had a higher RR genotype frequency (38.0 and 36.4 %, respectively) compared to controls (22.1 %). This result implies that both LDS and SDS may benefit from ACTN3 R allele existence, strengthening the notion of no clear ACTN3 polymorphism differences between power and endurance swimmers. Moreover, RR genotype and R allele frequency were higher among LDS (38.0 and 57.0 % for RR genotype and R allele frequency, respectively) compared to LDR (20.0 and 42.3 % for RR genotype and R allele frequency, respectively). This may be explained by differences in the specific activity duration of competitive swimming and running. The longest Olympic swim (1500 m ~ 15 min duration) is much shorter than the longest Olympic running race (marathon ~2 h 10-min duration), and therefore ACTN3 power characteristics are needed for long-distance swimmers. It is possible that if open-water swimmers (Olympic race—10K) had been included among the long-distance swimmers in the present study, the differences in the R allele frequency between LDS and LDR would disappear. When comparing the short-duration events, there is also a difference in the specific activity duration of competitive swimming and running. The 100-m distance covered in swimming at approximately 50 s, while the same distance covered in running at approximately 10 s. As a result, a sprint runner relies mostly on anaerobic energy sources, while a sprint swimmer uses also aerobic energy components. Therefore, the SDS and the LDS share some common features, and the ACTN3 RR genotype is not suitable as a distinguishing genetic marker between them, as it does for SDR and LDR.

## Conclusions

To conclude, the findings of the present study propose that while the ACTN3 R577X polymorphism is a genetic polymorphism that may distinguish between SDR and LDR, it cannot differentiate significantly between SDS and LDS. This suggests that the power of a specific genetic factor to serve as the sole divider between athletes of different specialties—aerobic or anaerobic—is limited. It seems that body physique, technique, tactics, and psychological factors are more likely to influence and determine such a distinction, especially in certain sport types, like swimming.
